# Exploring the Genetic Variability and Potential Correlations Between Nutritional Quality and Agro-Physiological Traits in Kabuli Chickpea Germplasm Collection (*Cicer arietinum* L.)

**DOI:** 10.3389/fpls.2022.905320

**Published:** 2022-07-01

**Authors:** Fatoumata Farida Traoré, Adil El-Baouchi, Youness En-nahli, Kamal Hejjaoui, Mohamed Louay Metougui, Aladdin Hamwieh, Quahir Sohail, Tawffiq Istanbuli, Said Boughribil, Moez Amri

**Affiliations:** ^1^Virology, Oncology, Biosciences, Environment and New Energies Laboratory, Department of Biology, Faculty of Science and Technology of Mohammedia, Hassan II University Mohammedia, Mohammedia, Morocco; ^2^Biodiversity and Plant Science Program, Department of AgroBioSciences, African Integrated Plant and Soil Research Group (AiPlaS), Mohammed VI Polytechnic University, Ben Guerir, Morocco; ^3^International Center for Agricultural Research in the Dry Areas (ICARDA), Rabat, Morocco; ^4^LPCMIO, Materials Science Center, Ecole Normale Supérieure, Mohammed V University of Rabat, Rabat, Morocco

**Keywords:** chickpea, grain yield, biofortification, nutritional quality, protein, micronutrients, macronutrients

## Abstract

Chickpea is an important source of plant-based protein and mineral elements such as iron (Fe) and zinc (Zn). The development of superior high-yielding germplasm with high nutritional value becomes central for any breeding program. Chickpea biofortified and nutrient-dense seeds can contribute to mitigate many human health problems associated with protein and micronutrients deficiency. In this study, 282 advanced chickpea lines were grown under field conditions to evaluate their agronomic performances and nutritional quality value. The trial was conducted under winter planting conditions during the cropping season 2017/2018 at ICARDA-Marchouch research station, Morocco. Results revealed high genetic variation and significant differences between the tested genotypes for all studied parameters. Under field conditions, the grain yield (GY) varied from 0.57 to 1.81 (t.ha^–1^), and 100-seed weight (HSW) ranged from 23.1 to 50.9 g. Out of the 282 genotypes, only 4 genotypes (i.e., S130109, S130058, S130066, and S130157) combined both good agronomic performances (GY, HSW) and high nutritional quality (protein, macronutrients, and micronutrients). Protein content ranged from 18.9 to 32.4%. For the whole collection, Fe content varied from 31.2 to 81 ppm, while Zn content ranged from 32.1 to 86.1 ppm. Correlation analysis indicated that the studied traits were significantly intercorrelated, with negative correlation between protein content and Zn concentration. Positive correlations were observed between grain filling time (F2M) and the micronutrients Zn, Cu, and Mn and macroelements K and Mg. Low positive correlation was also recorded between Pr and Fe concentrations. No significant correlation was observed between Fe and Zn. Positive correlations observed between main agronomic and nutritional quality traits makes easy any simultaneous enhancement when combining these traits.

## Introduction

Food and nutritional security are still far from being achieved, while hidden hunger remains the most prevalent challenge in many regions of the world (FAO, 2021). Currently, more than 820 million people still suffer from chronic undernourishment and malnutrition (FAO, 2020). At the same time, in the developing countries, the non-availability of high nutritional quality and nutrient-dense agricultural food products increases the prevalence of diseases, especially among poor population (Vandemark et al., 2018). Low consumption, poor diet diversity, and nutrient-poor food contribute to high levels of malnutrition. It was reported that malnutrition could negatively impact global gross domestic product by 10% per year (Hoddinott, 2016; FAO, 2020). Poor diets and nutrition cause not only death but also create huge economic burdens on healthcare with negative consequences on child development. Iron deficiency, for example, is usually associated with anemia, which is responsible for over 100,000 pregnancy deaths each year (Reifen, 2002). Iron deficiency during the fetal and neonatal period results in poor neurocognitive development of infants (Lozoff, 2007). Zinc deficiencies can cause fetal abnormalities as well as a dysfunction of the immune response leading to an increase in nonspecific infections (Solanki and Devi, 2020). Protein deficiencies have serious health consequences, such as atrophy and shrinkage of muscle tissue and immune system weakness (Khan et al., 2017). Protein deficiency is also the cause of stunted growth and development in infants and young children that can lead to permanent brain damage (Rajiv and Kawar, 2016). In addition, people suffering from protein deficiency are at high risk of developing abnormal blood coagulation (Rosendaal, 1999). To combat undernourishment and malnutrition, the development of nutrient-dense and biofortified germplasm could provide a sufficient range of food products necessary for a balanced diet available for poor households at affordable prices. There are opportunities to improve the sustainability of the food systems through the development of high nutritional quality and nutrient dense germplasm (Amri et al., 2019). Food legume crops could play a key role in improving food and nutritional security and building sustainable production systems due to their multiple agricultural and nutritional benefits. Pulses seeds contain 16–50% of the total human protein intake and contribute to almost one-third of the total protein nitrogen (Singh and Pratap, 2016). They provide two times as much protein as the major cereal crops (Asif et al., 2013). Chickpea, as one of the first domesticated legume crops (around 7,000 years ago), is the most commonly consumed legume crops globally (van der Maesen, 1987). With a global production of 14.25 million tons, chickpea is the third most important pulse crop in the world (Food Agriculture Organization, 2021). It is a key component of the main agriculture production systems, especially in South Asia and Africa. Chickpea is a very nutritious and inexpensive source of protein (23–24%) and reported to have a balanced amino acid composition (Muehlbauer and Sarker, 2017). Chickpea is also considered to be an ideal complement with cereals, which are reported to be rich in sulfur amino acids and poor in lysine (Wood and Grusak, 2007). Besides proteins, chickpea is also an important source of more than 15 micronutrients such as iron (Fe) and zinc (Zn) (Singh and Pratap, 2016; Amri et al., 2019). For more than four decades, national and international chickpea breeding programs have given more focus on improving disease and major abiotic stress resistance and increasing grain yield (Millán et al., 2015; Amri et al., 2019). Many high-yielding varieties carrying resistance to main diseases and environmental stresses have been developed (Amri et al., 2019). These improved varieties significantly contributed to enhancing chickpea productivity in many regions in the world (Amri et al., 2019). Most of the chickpea released varieties were developed either through conventional or molecular breeding, with main focus on agronomic performances through phenotypic selection under field or greenhouse conditions (Millán et al., 2015) and not much attention and research investment has been given to the seed nutritional quality improvement and biofortified germplasm development. Only recently, genetic biofortification and selection for high nutritional quality were considered as one of the main objectives of chickpea breeding programs at both national and international (CGIAR) levels. Biofortification and selection of nutrient-rich germplasm have become a priority area of research in food legumes in general and chickpea in particular. Therefore, the identification of high-yielding chickpea genotypes with high protein content and rich in micronutrients is necessary for the successful development of biofortified cultivars (Bhagyawant et al., 2015; Gupta et al., 2016). In this study, we aimed to explore the genetic variation and evaluating both agronomic and nutritional quality performances of 282 Kabuli chickpea improved lines in order to (i) identify superior high yielding germplasm with good nutritional quality and (ii) to explore potential correlations between agronomic traits and protein and micronutrient contents.

## Materials and Methods

### Plant Material and Field Trial Experiment

**FIGURE 1:**
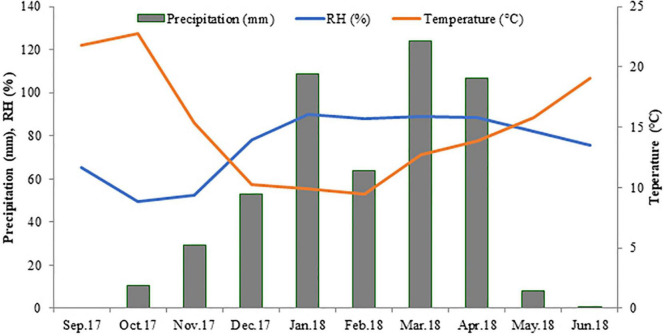
Precipitation (mm), relative humidity (RH, %) and average air temperature (°C) at Marchouch experimental station, Morocco, during the 2017–2018 cropping season.

A collection of 282 improved Kabuli chickpea advanced lines (F_8_) derived from crosses between parents carrying the most interested alleles (resistant to biotic and abiotic stresses). These crosses were performed at ICARDA, Terbol Research Station, Lebanon. The whole collection was subjected to field evaluation in order to assess their agronomic performances and nutritional quality value. The trial was conducted at ICARDA Marchouch research station (33.56°N, 6.63°W, 392 m altitude), Morocco, under winter planting during the cropping seasons 2017/2018 under rainfed conditions (weather parameters provided in Figure 1) using an alpha lattice design having two replications. Planting was carried out during the last week of December 2017 (winter planting). Each genotype was planted in 7.2 m^2^ plots (four rows of 3 m with 0.6 m inter-row spacing).

Different agronomic and phenophysiological parameters were recorded from plant emergence up to crop maturity using chickpea (*Cicer arietinum* L.) descriptors (International Board for Plant Genetic Resources, 1993). Both number of days to flowering (D2F) and number of days from flowering to maturity (F2M) were recorded for each plot from plant emergence to 50% flowering and from 50% plant flowering to 80% physiological maturity, respectively. Plant height (PH), highest low pod (HLP), biological yield (BY), grain yield (GY), harvest index (HI), and 100-seed weight (HSW) were all determined were all recorded at crop maturity and harvesting time.

### Nutritional Quality Assessment and Physicochemical Characterization of the Seeds

For the whole chickpea germplasm collection, the physicochemical parameters and seed weights were recorded on harvested seeds using the OPTO-Agri: TSW and Seed Biometry (Optomachines, 2022). The seeds were individually arranged in a transparent tray built in the OPTO-Agri. The weight was indicated immediately through an ultraprecise scale placed above the tray. The tray is illuminated by a high-resolution camera. The biometric data of the seeds (i.e., area, length, width, and perimeter) were obtained after digitization and image processing. Seeds’ area and weight were determined on 100 randomly selected seeds from each genotype/replication. The dry seeds were first scanned for seed area and weight and later soaked in 100 ml of distilled water for 24 h and then scanned again for the same traits. Hydration capacity (HC), weight variation (WV), and areas variation (AV) were determined for different tested genotypes. HC was calculated using the following equation (Williams et al., 1983):


HC=(S⁢S⁢W-S⁢D⁢W)/T⁢S⁢N

Where HC, hydration capacity (g.seed^–1^); SSW, soaked seed weight; SDW, dry seed weight (g); TSN, total seeds number.

### Protein Content Analysis

The total protein (Pr) was determined according to a modified Kjeldahl method (Baethgen and Alley, 1989). Chickpea flour was mineralized in sulfuric acid and salicylic acid solution in the presence of a catalyst (selenium). At the end of digestion, the amount of nitrogen was quantified using a colorimetric method followed by reading the absorbance in a T80 UV/VIS spectrophotometer using a wavelength of 650 nm. The crude protein content was obtained by multiplying the nitrogen content by a factor of 6.25. The whole set of collected data was then correlated with data recorded using the [near infrared spectroscopy (NIRS), FOSS-DS 2500]. High correlation values (*r* = 0.97) were observed using both analytic (Kjeldahl) and predicted NIRS data.

### Macro and Micronutrients Analysis

Major macronutrient and micronutrient contents were determined in the genotypes according to standard method developed by Pequerul et al. (1993) For each genotype, 500 mg of whole meal flour was placed in individual digestion tubes. A volume of 8 ml of 70% nitric acid (HNO_3_) was added and left to stand overnight. Each tube was then placed in a digestion block at 90°C for 1 h before adding 3 ml of 30% hydrogen peroxide (H_2_O_2_); the tubes were replaced again in the digestion block at 90°C until the complete digestion (colorless solution). The obtained extracts were filtered using Whatman papers (Grade 595) and then diluted to 1:10 with 6 M hydrochloric acid (HCl). The concentrations were measured by inductively coupled plasma–optical emission spectrometry (ICP-OES)–ICAP-7000-SERIES, Thermo Scientific at the Cereal and Legume Quality Laboratory, ICARDA, Morocco. The total content of macro-elements (K, P, Ca, and Mg) and micronutrients (Fe, Zn, Se, Mn, Cu, and Ni) were recorded.

### Statistical Analysis

The statistical analyses were performed using R software programs (version 4.1.2) and IBM SPSS 20.0 software program (IBM Corporation, New York, NY, United States). All statistical tests performed in this study were considered significant at *P* < 0.05. The analysis of variance (ANOVA) and Durkan’s test were studied using a linear mixed model with genotype as fixed factor and repetition and blocks as random factors. The Pearson’s correlations were used to assess the association between different parameters, including agronomic, physicochemical, and nutritional traits using prcomp function and mixOmics package. Hierarchical clustering analysis (HCA) was performed using the Ward’s method based on Euclidean distance, which is calculated after the standardization of the data to group chickpea accessions based on agronomic performance, and macronutrient and micronutrient compositions. Hierarchical clustering results were displayed graphically as tree diagrams using ggtree package in R studio. PCA and heatmap analysis were generated to determine, which traits explain most of variation as well as to determine genotype groups associated with major traits using ggplot2, factoextra, and FactoMineR packages in R studio.

## Results

### Field Evaluation of Agronomic Performances Under Winter Planting Conditions

All field parameters D2F, F2M, PH, HLP, BY, GY, HI, and HSW showed high variability (*P* ≤ 0.01) among the tested genotypes (Table 1). For the whole collection, D2F varied from 72.5 to 93 days, while F2M, from 36 to 56.5 days. BY ranged from 2 to 4.33 t.ha^–1^, while the GY varied from 0.57 (S130375) to 1.81 (S130157) t.ha^–1^. An average of 34.55 g was recorded for the HSW, which ranged from 23.1 (S130341) to 50.9 g (S130007). High heritability values were recorded for both D2F (0.88), F2M (0.69), and HSW (0.94) (Table 1).

**TABLE 1:** Range, mean, mean squares, *R*^2^ and heritability (h^2^) recorded for different agronomic parameters.

	Min.	Max.	Mean	Genotypes	*R* ^2^	Heritability (h^2^)
D2F	72.5 (S130004)	93.0 (S130217)	82.2	36.9**	0.89	0.88
F2M	36.0 (S130147)	56.5 (S130487)	45.2	42.6**	0.76	0.69
PH (cm)	38.5 (S130157)	68.5 (S130107)	53.6	59.4**	0.69	0.56
HLP (cm)	23.0 (S130230)	52.0 (S130197)	37.2	64.4**	0.70	0.61
BY (t.ha^–1^)	2.02 (S130430)	4.33 (S130129)	3.12	0.30**	0.66	0.49
GY (t.ha^–1^)	0.57 (S130375)	1.81 (S130157)	1.17	0.08**	0.68	0.54
HI	0.16 (S130375)	0.51 (S130008)	0.38	0.007**	0.67	0.51
HSW (g)	23.1 (S130341)	50.9 (S130007)	34.5	43.1**	0.95	0.94

Genotype effect was significantly different at **P < 0.01.

**FIGURE 2:**
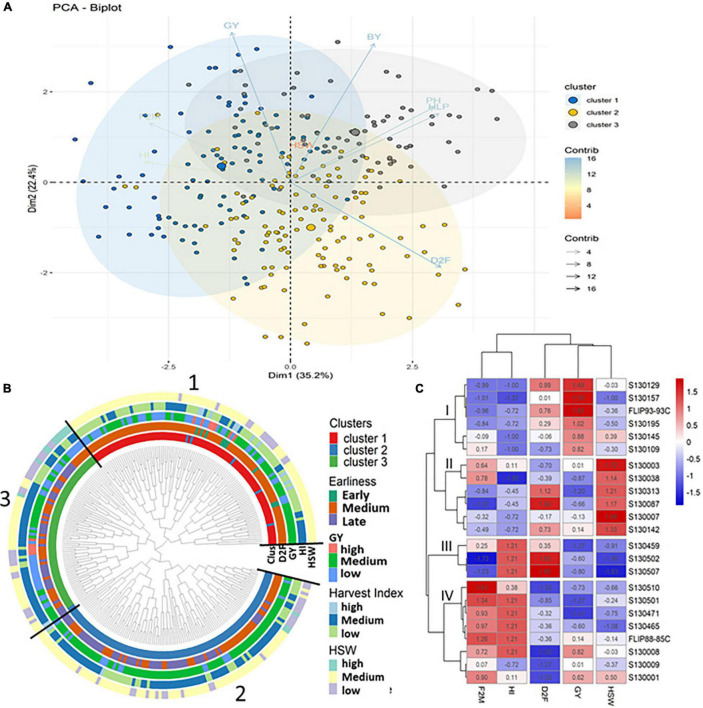
**(A)** Biplot of the first two dimensions of the principal component analysis (PCA). **(B)** Dendrogram showing the list of genotypes grouped in each cluster based on agronomic performances. **(C)** Heatmap and hierarchical clustering of 23 genotypes selected based on their agronomic performances.

**FIGURE 3:**
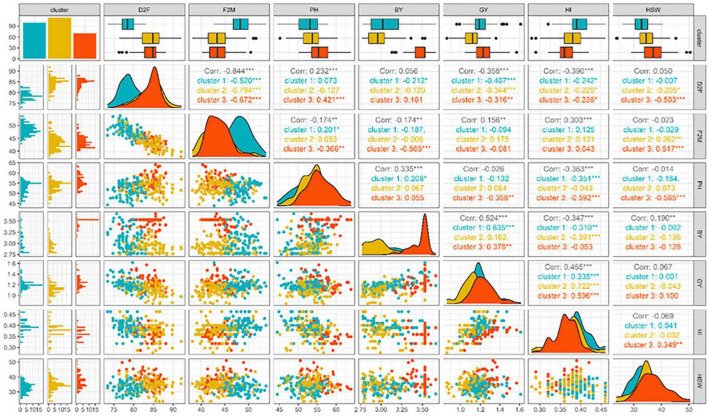
Pairwise correlations between different agro-morpho-physiological parameters recorded for all chickpea tested genotypes.

Based on agronomic parameters, both the principal component analysis (PCA) and cluster analysis revealed four major principal components (PC) coordinates (Table 2). The four first principal components, namely, PC1 (35.2%), PC2 (22.4%), PC3 (15.6%), and PC4 (12.4%) explained 85% of the total variation. The PC1 showed a high positive correlation with D2F (*r* = 0.74), PH (*r* = 0.70), and HLP (*r* = 0.73) against a negative correlation with F2M (*r* = –0.63) and HI (*r* = 0.72). PC2 was mainly and highly associated to BY (*r* = 0.73) and GY (*r* = 0.80) (Table 2). The 282 tested chickpea advanced lines could be clustered in three different groups presented in the PCA biplot (Figures 2A,B), namely, with 98, 112, and 72 genotypes each group. Cluster 1 consisted in early and extra-early flowering genotypes and relatively high GY and harvest index (HI). Cluster 2 mostly composed of genotypes with moderate values for all the agronomic parameters, while the third cluster included the most productive genotypes (GY), large seed size (high HSW), and high grain filling period (F2M). The pairwise correlation analysis of mean values (Figure 3) revealed negative correlations between D2F and GY for cluster 1 (*r* = –0.407***), cluster 2 (*r* = –0.344***), and cluster 3 (*r* = –0.316***). A negative significant correlation (*r* = –0.503***) was observed between D2F and HSW in cluster 3. For all three clusters, a high negative correlation (*r* = −0.844***) was also recorded between D2F and F2M. Positive and significant correlations were observed between F2M (*r* = 0.262**) and HSW (*r* = 0.517***) in cluster 2 and 3, respectively. Both cluster 1 and 3 showed also positive significant correlations between BY (*r* = 0.635***) and GY (*r* = 0.378***). For all clusters, positive correlation were observed between Hi and GY (*r* = 0.455***).

**TABLE 2:** Correlations and contribution of different agronomic parameters to the first three principal components of the principal component analysis (PCAs).

	PC1	PC2	PC3
D2F	**0.74**	−0.45	0.35
F2M	−**0.69**	0.31	−0.47
PH	**0.70**	0.40	−0.41
HLP	**0.73**	0.36	−0.41
BY	0.41	**0.73**	0.33
GY	−0.29	**0.80**	0.34
HI	−**0.72**	0.10	0.09
HSW	0.05	0.16	**0.57**

*Bold values for each column correspond to the parameters highly correlated (positive or negative) with each of the three PCs.*

The heat map and hierarchical clustering (Figure 2C) of 23 genotypes selected based on their performances for each of the studied traits/parameters including earlines (D2F), grain filling time (F2M), high GY, high harvest index (HI), and HSW show four distinct clusters. Genotypes with high GY were mainly grouped in cluster 1 and showed high to moderate D2F (S130129, S130195, and FLIP93-93C), while cluster 2 grouped together early genotypes with large seed size (S130313 and S130087). Cluster 4 grouped genotypes with high F2M and HI and moderate GY and HSW (S130001, S130501, S130471, S130465, S130008, and FLIP88-85C).

### Seed Physico-Chemical Characterization

Analysis of variance showed a highly significant differences (*P* ≤ 0.01) between genotypes for all physicochemical parameters (Table 3). Dry seed area ranged from 45.9 (S130341) to 73.1 mm^2^ (S130142), with an average of 59.4 mm^2^ recorded for all genotypes. An average single seed dry weight of 0.41 g was recorded for all genotypes varying from a minimum of 0.28 g for the genotype S130507 to a maximum of 0.54 g observed for the genotype S130160. Significant variations were recorded between dry and soaked seeds for all tested genotypes (Table 3). For the whole collection, average increases of 76.9 and 95.2% were observed for seed area and seed weight, respectively. The hydration capacity of the seeds varied considerably from 0.26 (S130507) to 0.54 g seed^–1^ (S130003), with a mean value of 0.39 g seed^–1^ for all tested genotypes. High heritability values were recorded for dry seed area (*r* = 0.90), dry seed weight (*r* = 0.90), and hydration capacity (*r* = 0.87).

**TABLE 3:** Range, mean, mean squares, and *R*^2^, and heritability (h^2^) of dry seed area, soaked seed area, area variation, dry seed weight, soaked seed weight, weight variation, and hydration capacity recorded for all tested chickpea genotypes.

	Min.	Max.	Mean	Genotypes	*R* ^2^	h^2^
Dry seed area (mm^2^)	45.9 (S130341)	73.1 (S130142)	59.4	46.5**	0.91	0.90
Soaked seed area (mm^2^)	79.5 (S130507)	132.4 (S130003)	105.1	149**	0.91	0.91
*Variation (%)*	–	–	**76.9**	**38.6** **	**0.55**	
Dry seed weight (g)	0.3 (S130507)	0.54 (S130160)	0.41	0.01**	0.91	0.90
Soaked seed weight (g)	0.5 (S130507)	1.08 (S130007)	0.80	0.02**	0.92	0.91
*Variation (%)*	–	–	**95.2**	**44.2** *	**0.63**	
HC (g.seed^–1^)	0.26 (S130507)	0.54 (S130003)	0.39	0.005**	0.88	0.87

Genotype effect was significantly different at *P < 0.05, **P < 0.01, respectively.

*Bold values for each column correspond to the parameters highly correlated (positive or negative) with each of the three PCs.*

### Nutritional Quality Assessment, Protein, Micro, and Macronutrients Content

**FIGURE 4:**
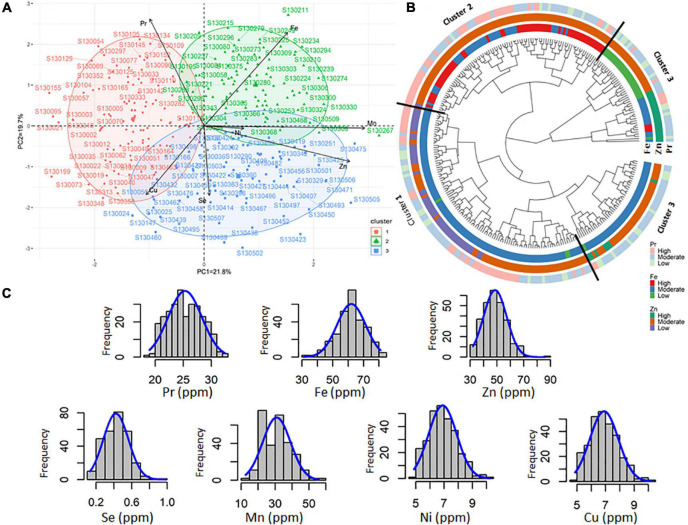
**(A)** Biplot of the first two dimensions of the PCA. **(B)** Dendrogram showing the list of genotypes grouped in each cluster with the level of micronutrients. **(C)** Normal distribution and frequency different tested genotypes and their content in protein (Pr), concentrations of iron (Fe), zinc (Ze), selenium (Se), manganese (Mn), nickel (Ni), and copper (Cu).

Analysis of variance revealed significant (*P* ≤ 0.01) variation among tested genotypes for micronutrients content except for selenium (Se) and nickel (Ni) (Table 4). Protein content (Pr) ranged from 18.9% (S130425) to 32.4% (S130155), with an average of 25.31%. For all tested genotypes mean values of 62 ppm and 48.6 ppm were observed for iron (Fe) and zinc (Zn), respectively. Fe content ranged from a minimum of 31.2 (S130462) to a maximum of 81 ppm (S130373), while Zn contents ranged from 32.1 (S130073) to 86.1 ppm (S130342). Manganese (Mn) content varied from 12.6 (S130313) to 55.7 ppm (S130274). Copper (Cu), selenium (Se), and nickel (Ni) were found in lower amounts, ranging from 4.86 to 10.2 ppm, 0.13 to 0.98 ppm, and 0.08 to 0.93 ppm, respectively. Genotypes S130024, S130419, and S130481 showed the highest values of Cu (10.2 ppm), Se (0.98 ppm), and Ni (0.93 ppm) content, respectively. Low heritability values were recorded for micronutrients contents except for Zn content (0.59) which had moderate heritability. PCA based on protein and micronutrient contents showed that the first two components PC1 (21.8%) and PC2 (19.7%) explained together 41.5% of the total genotypic variation (Figure 4A). PC1 was positively and strongly correlated with Mn (*r* = 0.79) and Zn (*r* = 0.72) but negatively correlated with protein (*r* = –0.26) and Cu (*r* = –0.28), while PC2 was positively correlated with Pr (*r* = 0.71) and Fe (*r* = 0.62) content (Table 5). The PCA biplot (Figures 4A,B) showed that all tested genotypes could be grouped into three different clusters with 106, 89, and 87 genotypes each.

**TABLE 4:** Range, mean and mean squares, *R*^2^, and heritability (h^2^) of major macro- and micronutrients for all tested chickpea genotypes.

	Min.	Max.	Mean	Genotypes	*R* ^2^	Heritability (h^2^)
Fe	31.2 (S130462)	81.0 (S130373)	62.0	174.45**	0.56	0.24
Zn	32.1 (S130073)	86.1 (S130342)	48.6	149.93**	0.7	0.59
Se	0.13 (S130324)	0.98 (S130419)	0.42	0.04	0.53	0.19
Mn	12.6 (S130313)	55.7 (S130274)	30.9	136.74**	0.63	0.05
Ni	0.08 (S130004)	0.93 (S130481)	0.33	0.053**	0.60	0.37
Cu	4.86 (S130415)	10.2 (S130024)	6.9	2.02	0.52	0.12
K	6130.8 (S130120)	13476.4 (S130505)	8789.6	2146506.4	0.50	0.08
P	1289.2 (S130371)	3606.4 (S130318)	2255.4	375273	0.50	0.05
Ca	729.8 (S130019)	1848.2 (S130295)	1113.7	79682.4	0.48	0.02
Mg	785.7 (S130396)	1574.0 (S130257)	1205.7	26963.5	0.53	0.25

Genotype effect was significantly different at *P < 0.05, **P < 0.01, respectively.

**TABLE 5:** Correlations and contribution of protein content and macronutrient and micronutrient concentrations to the first three principal components of the principal component analysis (PCAs).

	PC1	PC2	PC3
Pr	−0.2698	**0.7125**	0.1411
Fe	**0.4148**	**0.6225**	−**0.4091**
Zn	**0.7235**	−0.2367	0.2170
Se	0.0256	−**0.4635**	−**0.5781**
Mn	**0.7980**	−0.0143	−0.0714
Ni	0.1976	−0.0162	**0.7812**
Cu	−0.2875	−**0.4582**	0.1107
K	**0.6631**	**0.4357**	−0.2993
P	−**0.747**	0.1788	0.3082
Ca	0.0835	**0.8449**	**0.4269**
Mg	**0.5578**	−**0.405**	**0.7046**

*Bold values for each column correspond to the parameters highly correlated (positive or negative) with each of the three PCs.*

The first cluster consisted of genotypes with high Pr content and moderate Zn and Fe values. The second cluster contained genotypes with high Fe and Pr contents and low Zn content, while the third cluster grouped genotypes with high Zn.

The whole tested genotypes collection showed a normal distribution frequency for Pr and all micronutrients content (Figure 4C). Results showed also a large variation among all chickpea tested genotypes for the studied macronutrients; potassium (K), phosphorus (P), calcium (Ca), and magnesium (Mg). For all genotypes, K was the most abundant element ranging from 6,130.8 (S130120) to 13,476.4 ppm (S130505), followed by P, Ca, and magnesium (Mg) (Table 4). P content ranged from 1,289.2 (S130371) to 3,606.4 mg kg^–1^ (S130318). The highest Ca content was recorded for the genotype S130295 (1,848.21 ppm), against a minimum of 729.83 ppm observed for the genotype S130019. The Mg content varied from 785.73 (S130396) to 1,574.03 ppm (S130257).

**FIGURE 5:**
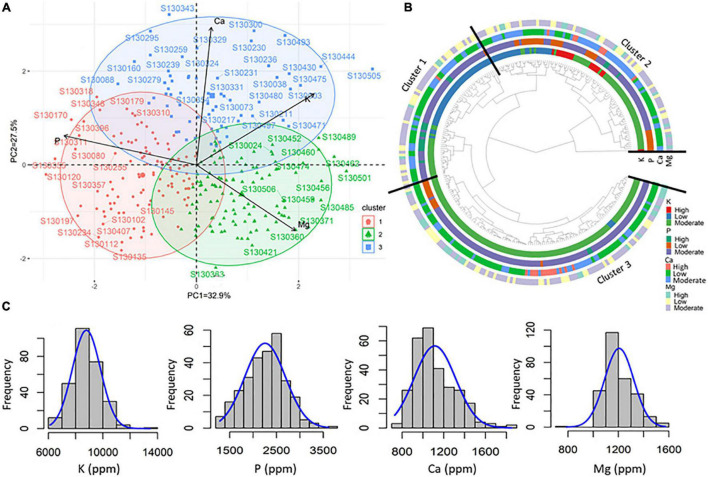
**(A)** Biplot of the first two dimensions of the PCA. **(B)** Dendrogram showing the list of genotypes grouped in each cluster with the level of major macronutrients. **(C)** Normal distribution and frequency different tested genotypes and their contents in potassium (K), phosphorus (P), calcium (Ca), and magnesium (Mg).

Principal component analysis showed that the first two components PC1 (32.9%) and PC2 (22.5%) explained together 55.4% of the total genotypic variation (Figure 5A). The PC1 showed a high positive correlation with high Mg (*r* = 0.55) and K (*r* = 0.66) and a negative correlation with P (*r* = –0.74), while PC2 was mainly and highly associated with high Ca (*r* = 0.84) (Table 5). PCA biplot and ggtree (Figures 5A,B) showed that all tested genotypes could be grouped into three different clusters with 109, 107, and 66 genotypes each. Cluster 1 grouped genotypes with high P concentration. The second cluster grouped genotypes with high K concentration and low *P*-values. The third cluster grouped genotypes with high Ca and Mg concentrations. For all studied macronutrients, i.e., K, P, Ca, and Mg, results showed a normal distribution frequency of all tested genotypes (Figure 5C).

**FIGURE 6:**
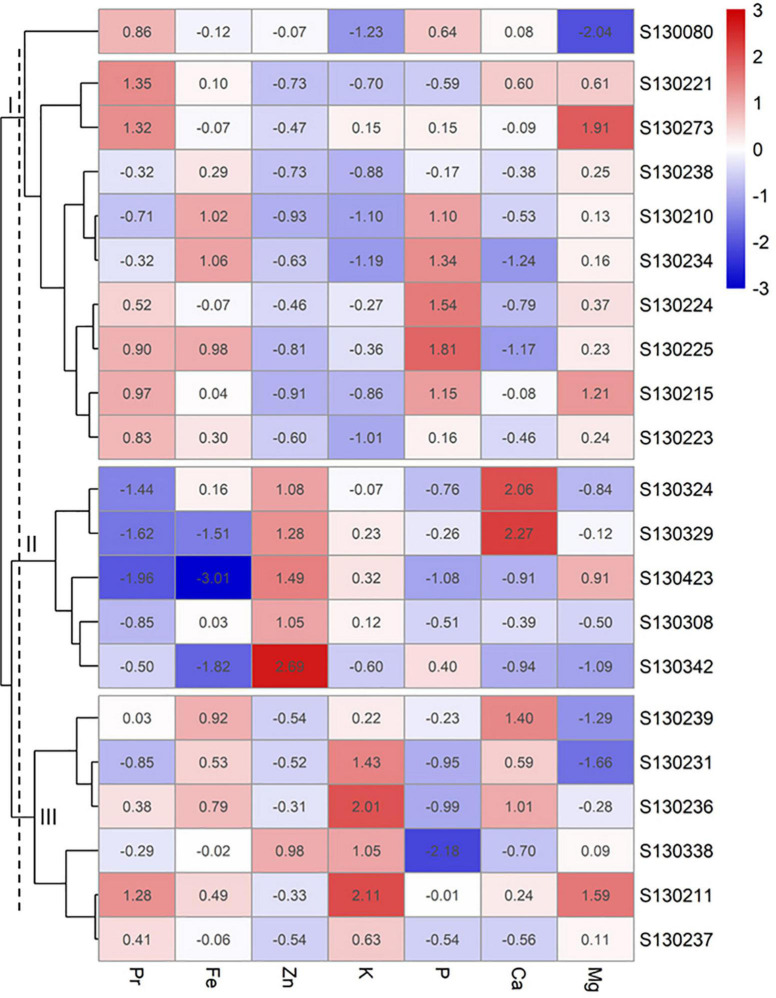
Heatmap and hierarchical clustering of 21 genotypes selected based on protein and major micronutrients (Fe and Zn) and macroelements (K, P, Ca, and Mg).

The heat map and hierarchical clustering (Figure 6) of 21 genotypes selected for their high content on protein (Pr) and six macronutrients (K, P, Mg, and Ca) and micronutrients (Fe and Zn) revealed three different clusters. The first group consisted in 10 genotypes with moderate to relatively high Pr, Fe, and P such as S130225, S130223, S130080, and S130215. The second cluster grouped together 5 genotypes with high Zn and Ca such as S130324 and S130329. The last and third cluster consisted in 6 genotypes with moderate Pr, Fe Zn, and Ca values and high K content such as S130236, S130211, and S130237.

### Correlations Between Agronomic and Nutritional Quality Parameters

**FIGURE 7:**
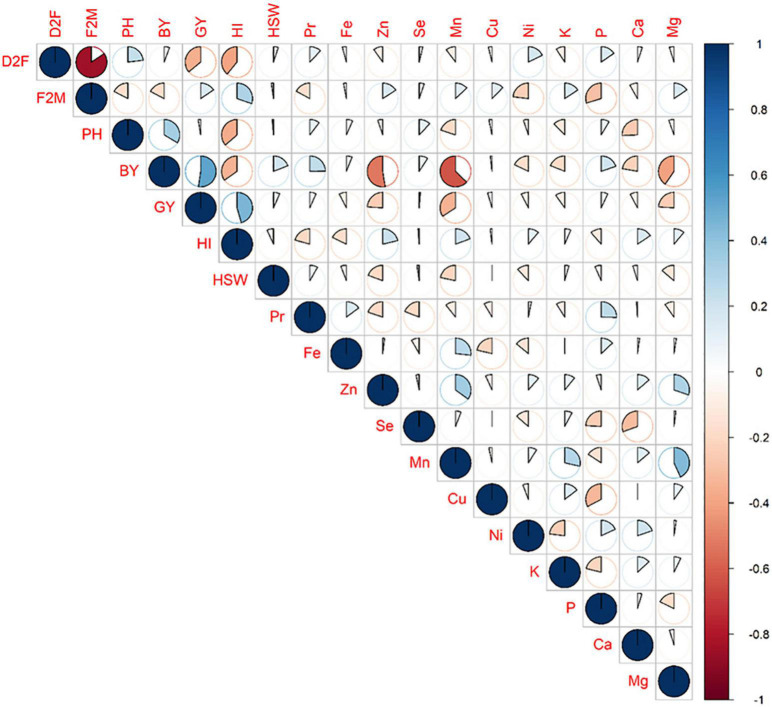
Pearson matrix and correlations between agro-physiological and nutritional quality parameters. Positive correlations are displayed in blue and negative correlations in red. The spot size and color intensity are proportional to the correlation coefficients.

**FIGURE 8:**
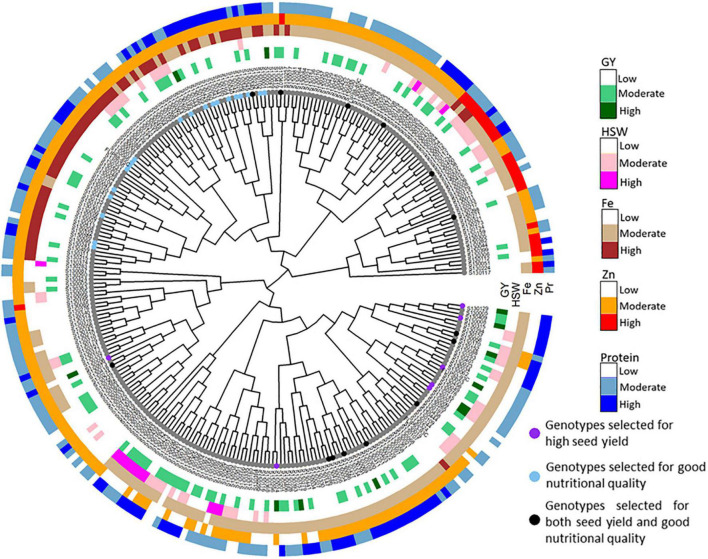
Dendrogram showing the total list of tested genotypes grouped in different clusters generated based on agronomic performances (GY and HSW) and nutritional quality traits (protein, Fe, and Zn contents).

Pearson correlation analysis was carried out (Figures 7, 8) to explore any potential associations between agro-physiological parameters and nutritional quality traits. Results showed a relatively high negative correlation between BY and Zn (*r* = –0.53) and Mn (*r* = –0.63) concentrations. Likewise, negative correlation were also recorded between GY and Zn (*r* = –0.24), Mn (*r* = –0.34), and Mg (*r* = –0.24) concentrations. HSW was also negatively correlated with Zn (*r* = –0.19) and Mn (*r* = –0.22) concentrations. Pr content and both Fe and Se showed no significant correlations with both GY and HSW. Results revealed also low positive correlations between grain filling time (F2M) and the micronutrients Zn (*r* = 0.16), Cu (*r* = 0.13), Mn (*r* = 0.12), and both macro-elements K and Mg (*r* = 0.16). Low positive correlation was also recorded between Pr and Fe contents (*r* = 0.15), whereas it was negatively correlated with Zn (*r* = –0.20) and Se (*r* = –0.19).

The superimposition of both major agronomic parameters GY and HSW with Pr, Fe, and Zn contents was used to cluster all 282 tested genotypes (Figure 7). The dendrogram was used to identify and select the best genotypes combining both agronomic performances and good nutritional quality. Clustering analyses allowed to identify and select three distinct groups of genotypes based on their agronomic performances and nutritional quality value (Figure 8). The first group with genotypes all genotypes showing high GY and HSW. The second group consisted in good quality genotypes with high Pr [S130221 (30.1%), S130273 (30%), S130211 (29.9%)], Fe [S130249 (80.9 ppm), S130296 (79 ppm), S130309 (79.5 ppm), S130225 (77.6 ppm)], and Zn [S130342 (86.1 ppm), S130423 (71.8 ppm), S130329 (69.3 ppm), and S130407 (68.7 ppm)]. The third group consisted on genotypes with good agronomic performances and high nutritional quality such as S130109, S130058, S130066, and S130157. Finally, among all tested genotypes, 10 genotypes were selected for their good agronomic performances and high nutritional quality value. S130221 and S130273 stood out for their high protein content; S130234 and S130210 for high Fe concentration and S130342 and S130423 for high Zn. In addition, based on agronomic performances (Figure 2C), early flowering genotypes (S130510 and S130008) and genotypes with high grain yield (S130157 and FLIP93-93C) were identified.

## Discussion

Protein and micronutrient deficiency especially Fe and Zn pose a serious problem for human health. Selection and development of high nutritional value germplasm can significantly contribute in overcoming this problem by providing an easy access to biofortified and nutrient-dense genotypes. In this study, we evaluated the agronomic performance and nutritional quality value of 282 advanced chickpea lines. Results showed a large genetic variation and revealed a significant differences between the studied genotypes for all agronomic and nutritional quality traits. Most of the agronomic traits showed high heritability, especially the D2F (0.88) and HSW (0.94). Superior genotypes with good agronomic performances and high nutritional value were identified such as S130109, S130058, S130066, and S130157. Similar results were reported in previous studies for HSW (Ali and Ahsan, 2012), D2F (Khan et al., 2011), and GY (Malik et al., 2009). Besides their high GY potential, earlines and large seed size, which is an important market trait for Kabuli chickpea, were characterized by high protein, Fe, and Zn concentrations. Usually, varieties with large seed size (>40 g) have better market price as are preferred by consumers especially in the Middle East and North African (MENA) region. In addition, significant correlations were recorded between GY and D2F and F2M. Earliness expressed by low D2F was positively correlated with GY, which shows that early flowering has a positive effect on grain yield because it prolongs the reproductive phase and leads to a comparatively longer seed filling period (F2M). Same results have been reported by Kumar and Abbo (2001) and Sundaram et al. (2019) on chickpeas. In contrast Gaur et al. (2015b), suggested that, in general, the early flowering genotypes also mature early and the early flowering does not result in extending of the reproductive period under residual soil moisture conditions. Mbarek et al. (2013) have reported an indirect negative correlation between grain yield and early flowering and maturity through a modeling study based on a pot experiment conducted under controlled conditions on chickpeas genotypes. Other studies have also reported negative significant correlations between grain yield and flowering time on lentils (Vandemark et al., 2018) and faba bean genotypes (Alghamdi, 2007).

In addition to agronomic performances and with the growing interest in chickpea consumption due to their high nutritional value and health benefits, breeders started to give more emphasis to nutritional quality traits. In our study, negative correlation was recorded between the two main agronomic parameters GY and HSW and both micronutrients Zn and Mn contents, but no correlation was recorded with protein and Fe contents. It turns out that chickpea small seeds are more rich in the two micronutrients Zn and Mn compared with large seeds. These results are in line with what was reported by Vandemark et al. (2018) who found a negative correlation between Zn content and chickpea GY and seed size. Moreover, negative correlation between GY and zinc concentration has previously been reported in studies on lentil (Vandemark et al., 2018) soybeans (Gibson and Mullen, 2001), and peas (Ma et al., 2017). Vandemark et al. (2018) suggested that negative correlations between GY and mineral concentrations are due to a dilution effect, whereby the whole-plant uptake or internal pool of certain minerals may be limited and unable to meet the genetic potential of a higher seed mineral load. Hence, breeding for increased uptake of some minerals may be needed to sustain higher mineral concentrations in higher-yielding lines (Sankaran and Grusak, 2014).

The non-correlation recorded between GY/HSW and protein/Fe contents observed in this study was different from what was reported by Khattak et al. (2006) who found a positive correlation between protein content and both GY and HSW. Vandemark et al. (2018) recorded positive correlations between Fe and GY in chickpea and lentils. In contrast, some researchers have recorded negative correlations between GY and protein content in pulses like chickpea (Frimpong et al., 2009). Negative correlations were also observed between protein content and HSW in chickpea (Falco et al., 2010) and barley (Bouhlal et al., 2021). Our results showed also a wide range for different nutritional quality traits among the tested genotypes collection. A large variation was recorded for protein content as well as macronutrients and micronutrients. Such variation was also reported by Sharma et al. (2013) who found also a considerable varietal differences in Fe and Zn concentrations in Indian chickpea cultivars. In our study, large variation was observed especially for protein (18.9–32.4%), Fe (31.2–81 ppm), Zn (32.1–86.1 ppm), and Se (0.13–0.98 ppm). Such variability is very important and can be considered as a good source for chickpea nutritional quality improvement in breeding programs. The maximum concentration levels observed for Fe, Zn, and Se in our tested collection exceed those reported by Thavarajah and Thavarajah (2012) who reported respective maxima of 67, 74, and 0.56 ppm. In addition, most of the tested genotypes (>50%) have high protein content exceeding 25%. Some of these genotypes exceeded the maximum level of protein content previously reported in chickpea (Wood and Grusak, 2007; Özer et al., 2010). Furthermore, previous studies on chickpea reported high heritability for both Fe and Zn (Sab et al., 2020; Tefera, 2021), which is in contrast with our study. Our results showed moderate and low heritability for both Zn (59%) and Fe (24%) concentrations.

A contrasting correlations between protein content, and both Fe and Zn concentrations were observed in this study. A slight positive correlation was recorded between Pr and Fe (*r* = 0.150) against a negative correlation recorded between Pr and Zn (*r* = –0.200). The positive correlation between protein and Fe contents in chickpea was also reported by Patane (2006). The positive correlation between protein and Fe contents in chickpea makes easy and suggests that these two elements could be improved simultaneously. Regarding the correlation between protein and Zn concentrations, similar results were also reported by Kaya et al. (2009) on chickpea. Such negative correlation suggests that the high crude protein content of chickpea seeds may lead to decreased Zn concentration, which could be explained by the fact that zinc plays an important role in protein synthesis in plants (Hajiboland and Amirazad, 2010). The negative correlation between protein and Zn was different from what was mentioned by Kutman et al. (2010) who reported a significant positive correlation between wheat grain concentrations of Zn and total nitrogen. Same authors mentioned based on seed staining studies that protein and Zn were co-translocated in the wheat grain.

For macronutrients, K was the most abundant macronutrient present in chickpea flour, followed by P, Ca, and Mg. Similar conclusions were made by Zia-Ul-Haq et al. (2007). The macronutrient concentration levels observed for the tested chickpea collection were lower than those reported in previous studies conducted on chickpea (Marioli Nobile et al., 2013; Gaur et al., 2015a). Other studies conducted on a Spanish and Pakistani lentil collection showed lower levels of macronutrients than those encountered in the present chickpea collection (Zia-Ul-Haq et al., 2011; Plaza et al., 2021). Important positive correlations were recorded between Mg and both micronutrients Mn (*r* = 0.430) and Zn (*r* = 0.300) against negative correlations observed between Se and both Ca (*r* = 0.310) and P (*r* = 0.240). For the same species, Vandemark et al. (2018) reported positive correlations between P and both Zn and K and Mg and Fe. Some of these positive correlations between macronutrients and micronutrients could be explained by existing of similar pathways and/or transporters controlling the uptake, use, and translocation in the grain of these elements. In addition, the positive correlation between seed protein content and P concentration is related to the inherent relationship between the phosphorus and protein content in the grain (Tsai et al., 2018). Phosphorus is abundant in proteins (phosphoprotein); some protein amino acids interact with the phosphate anion, either in its inorganic state or bound in a nucleotide fragment (Gruber et al., 2014).

**FIGURE 9:**
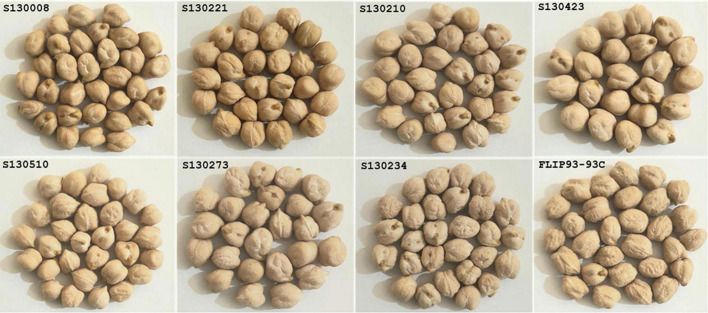
Top two lines identified for early flowering (S130510 and S130008), high gain yield (FLIP93-93C), high protein content (S130221 and S130273), high Fe (S130234 and S130210), and high Zn (S130423) concentrations.

The identification of genotypes with good agronomic performance (high GY and HSW) and good nutritional quality is the main objective of breeding programs. Most of the studied parameters showed positive and negative correlations with each other, which make complicated the possibility to combine all different agronomic and quality traits in one superior genotype. From breeding perspective, it would be better to focus on the main traits with positive correlations. If two traits are favorably correlated, selection can simultaneously improve both by tandem selection, indirect selection, or a trait index (Bernardo, 2010). Moreover, the identification of protein-rich and nutrient-dense chickpea germplasm could help breeders to select donors for targeted protein, Fe, and Zn biofortification. Genotypes with both good agronomic performance and high nutritional quality (S130109, S130058, S130066, and S130157) have also been identified (Figure 9). These selected genotypes can be used as quality trait donors in breeding programs for the development of high-yielding biofortified varieties.

## Conclusion

Chickpea is one of the most consumed legume crops worldwide. Selection of high yielding and biofortified chickpea germplasm could significantly contribute in meeting food and nutritional security. This study was conducted to assess agronomic performances and nutritional quality value of a Kabuli chickpea advanced lines collection. The study revealed high and significant genetic variation among all tested genotypes for both agro-physiological and nutritional quality traits. Around 28 genotypes representing 10% of the total collection showed high yield potential and large seed size with an HSW exceeding 40 g. Interesting levels of protein content have been revealed. Half of the genotypes (50.7%) showed a protein content above 25%. Significant levels of iron and zinc (greater than 60 ppm) were also observed. Genotypes (S130109, S130058, S130066, and S130157) combining both good agronomic performance and good nutritional quality were selected. The chickpea genotypes selected in this research may be useful as parent material for developing improved chickpea cultivars with desirable traits.

## Data Availability Statement

The raw data supporting the conclusions of this article will be made available by the authors, without undue reservation.

## Author Contributions

MA, SB, and FF designed the research. MA, AH, KH, and TI provided the chickpea germplasms from ICARDA. FF, AE-B, KH, and MA performed the experiments. FF, AH, KH, AE-B, YE, MM, and MA contributed to materials and analysis tools. FF and MA wrote and drafted the manuscript. AE-B, YE, MM, AH, QS, SB, and MA revised the manuscript. All authors approved the final manuscript.

## Conflict of Interest

The authors declare that the research was conducted in the absence of any commercial or financial relationships that could be construed as a potential conflict of interest.

## Publisher’s Note

All claims expressed in this article are solely those of the authors and do not necessarily represent those of their affiliated organizations, or those of the publisher, the editors and the reviewers. Any product that may be evaluated in this article, or claim that may be made by its manufacturer, is not guaranteed or endorsed by the publisher.

## References

[B1] Alghamdi S. (2007). Genetic behavior of some selected faba bean genotypes. *Afr. Crop Sci. Conf. Proce.* 8 709–714.

[B2] Ali Q. Ahsan M. (2012). Estimation of genetic variability and correlation analysis for quantitative traits in chickpea (*Cicer arietinum* L.). *Int. J. Agro Vet. Med. Sci.* 6 241–249.

[B3] Amri M. Niane A. A. Agrawal S. K. Kemal S. A. Hamwieh A. Amri A. (2019). Principales activités des programmes d’amélioration génétique de la lentille et du pois chiche Kabuli à ICARDA. *Innov. Agron.* 74:15. 10.15454/5V70WM

[B4] Asif M. Rooney L. W. Ali R. Riaz M. N. (2013). Application and opportunities of pulses in food system: a review. *Crit. Rev. Food Sci. Nutr.* 53 1168–1179. 10.1080/10408398.2011.574804 24007421

[B5] Baethgen W. Alley M. (1989). A manual colorimetric procedure for measuring ammonium nitrogen in soil and plant kjeldahl digests. *Commun. Soil Sci. Plant Anal.* 20 961–969. 10.1080/00103628909368129

[B6] Bernardo R. N. (2010). *Breeding for Quantitative traits in Plants.* Woobury, NY: Stemma Press.

[B7] Bhagyawant S. S. Gupta N. Shrivastava N. (2015). Biochemical analysis of chickpea accessions vis-a-vis; zinc, iron, total protein, proline and antioxidant activity. *Am. J. Food Sci. Technol.* 3 158–162.

[B8] Bouhlal O. Affricot J. R. Puglisi D. El-Baouchi A. El Otmani F. Kandil M. (2021). Malting quality of ICARDA elite winter barley (*Hordeum vulgare* L.) germplasm grown in moroccan middle atlas. *J. Am. Soc. Brew. Chem.* 1–12. 10.1080/03610470.2021.1978036

[B9] Falco E. Imperato R. Landi G. Nicolais V. Piccinelli A. Rastrelli L. (2010). Nutritional characterization of *Cicer arietinum* L. cultivars with respect to morphological and agronomic parameters. *Emir. J. Food Agric.* 22:377. 10.9755/ejfa.v22i5.4825

[B10] FAO (2020). *The State of Food Security and Nutrition in the World 2020.* Rome: Food and Agriculture Organization of the United Nations. 10.4060/CA9692EN

[B11] FAO (2021). *The State of Food Security and Nutrition in the World 2021.* Rome: Food and Agriculture Organization of the United Nations. 10.4060/CB4474EN

[B12] Food and Agriculture Organization (2021). *FAOSTAT Données de L’Alimentation et de L’Agriculture.* Available online at: http://www.fao.org/faostat/fr/ (accessed September 15, 2021).

[B13] Frimpong A. Sinha A. Tar’an B. Warkentin T. D. Gossen B. D. Chibbar R. N. (2009). Genotype and growing environment influence chickpea (*Cicer arietinum* L.) seed composition. *J. Sci. Food Agric.* 89 2052–2063. 10.1002/jsfa.3690

[B14] Gaur P. M. Samineni S. Tripathi S. Varshney R. K. Gowda C. L. L. (2015a). Allelic relationships of flowering time genes in chickpea. *Euphytica* 203 295–308.

[B15] Gaur P. M. Samineni S. Sajja S. Chibbar R. N. (2015b). Achievements and challenges in improving nutritional quality of chickpea. *Legum. Res.* 9 31–33. 10.1016/j.foodhyd.2020.106565 33941996 PMC7859705

[B16] Gibson L. R. Mullen R. E. (2001). Mineral concentrations in soybean seed produced under high day and night temperature. *Can. J. Plant Sci.* 81 595–600.

[B17] Gruber M. Greisen P. Junker C. M. Hélix-Nielsen C. (2014). Phosphorus binding sites in proteins: structural preorganization and coordination. *J. Phys. Chem. B* 118 1207–1215. 10.1021/jp408689x 24404899

[B18] Gupta D. S. Thavarajah D. McGee R. J. Coyne C. J. Kumar S. Thavarajah P. (2016). Genetic diversity among cultivated and wild lentils for iron, zinc, copper, calcium and magnesium concentrations. *Aust. J. Crop Sci.* 10 1381–1387. 10.21475/ajcs.2016.10.10.pne6

[B19] Hajiboland R. Amirazad F. J. P. (2010). Growth, photosynthesis and antioxidant defense system in Zn-deficient red cabbage. *Plants Soil Environ.* 56 209–217.

[B20] Hoddinott J. (2016). *Global Panel on Agriculture and Food Systems for Nutrition Working Paper.* London: Food systems, 21.

[B21] International Board for Plant Genetic Resources (1993). *Descriptors for Chickpea (Cicer arietinum* L.). Rome: IBPGR, 31.

[B22] Kaya M. Küçükyumuk Z. Erdal I. (2009). Phytase activity, phytic acid, zinc, phosphorus and protein contents in different chickpea genotypes in relation to nitrogen and zinc fertilization. *Afr. J. Biotechnol.* 8 4508–4513.

[B23] Khan A. Khan S. Jan A. A. Khan M. (2017). Health complication caused by protein deficiency. *J. Food Sci. Nutr.* 1 1–2. 10.35841/aajfsn.1000101

[B24] Khan R. Farhatullah Khan H. (2011). Dissection of genetic variability and heritability estimates of chickpea germplasm for various morphological markers and quantitative traits. *Sarhad J. Agric.* 27 67–72.

[B25] Khattak A. B. Khattak G. S. S. Mahmood Z. Bibi N. Ihsanullah I. (2006). Study of selected quality and agronomic characteristics and their interrelationship in kabuli-type chickpea genotypes (*Cicer arietinum* L.). *Int. J. Food Sci. Tech.* 41 1–5. 10.1111/j.1365-2621.2006.01193.x

[B26] Kumar J. Abbo S. (2001). Genetics of flowering time in chickpea and its breeding on productivity in semiarid environments. *Adv. Agron.* 72 107–137. 10.3389/fpls.2017.01140 28729871 PMC5498527

[B27] Kutman U. B. Yildiz B. Ozturk L. Cakmak I. (2010). Biofortification of durum wheat with zinc through soil and foliar applications of nitrogen. *Cereal Chem.* 87 1–9. 10.1094/CCHEM-87-1-0001

[B28] Lozoff B. (2007). Iron deficiency and child development. *Food Nutr. Bull.* 28 S560–S571. 10.1177/15648265070284S409 18297894

[B29] Ma Y. Coyne C. J. Grusak M. A. Mazourek M. Cheng P. Main D. (2017). Genome-wide SNP identification, linkage map construction and QTL mapping for seed mineral concentrations and contents in pea (*Pisum sativum* L.). *BMC Plant Biol.* 17:43. 10.1186/s12870-016-0956-4 28193168 PMC5307697

[B30] Malik S. R. Bakhsh A. Asif M. A. Iqbal U. Iqbal S. M. (2009). Assessment of genetic variability and interrelationship among some agronomic traits in chickpea. *Int. J. Agric. Biol.* 12:6.

[B31] Marioli Nobile C. G. Carreras J. Grosso R. Inga M. Silva M. Aguilar R. (2013). Proximate composition and seed lipid components of “kabuli”-type chickpea (&i&*Cicer arietinum*&/i& L.) from Argentina. *Agric. Sci.* 4 729–737. 10.4236/as.2013.412099

[B32] Mbarek K. B. Boubaker M. Hannachi C. (2013). Modélisation du rendement grain du pois chiche (*Cicer arietinum* L.) du type «kabuli» sous les conditions édapho-climatiques du semi aride supérieur tunisien. *Rev. Mar. Sci. Agron. Vét.* 2 37–49.

[B33] Millán T. Madrid E. Cubero J. I. Amri M. Castro P. Rubio J. (2015). “Chickpea,” in *Grain Legumes. Handbook of Plant Breeding*, ed. De Ron A. M. (New York, NY: Springer New York), 85–109. 10.1007/978-1-4939-2797-5_3

[B34] Muehlbauer F. J. Sarker A. (2017). “Economic importance of chickpea: production, value, and world trade,” in *The Chickpea Genome. Compendium of Plant Genomes*, eds Varshney R. K. Thudi M. Muehlbauer F. (Cham: Springer International Publishing), 5–12. 10.1007/978-3-319-66117-9_2

[B35] Optomachines (2022). *OPTO-Agri: TSW & Seed Biometry.* Available online at: https://optomachines.fr/home/agronomics/opto-agri/ (accessed November 9, 2021).

[B36] Özer S. Karaköy T. Toklu F. Baloch F. S. Kilian B. Özkan H. (2010). Nutritional and physicochemical variation in Turkish kabuli chickpea (*Cicer arietinum* L.) landraces. *Euphytica* 175 237–249.

[B37] Patane C. (2006). Variation and relationships among some nutritional traits in sicilian genotypes of chickpea (*Cicer arietinum* L.). *J. Food Qual.* 29 282–293. 10.1111/j.1745-4557.2006.00074.x

[B38] Pequerul A. Pérez C. Madero P. Val J. Monge E. (1993). “A rapid wet digestion method for plant analysis,” in *Optimization of Plant Nutrition*, eds Fragoso M. A. C. Van Beusichem M. L. Houwers A. (Dordrecht: Springer), 3–6.

[B39] Plaza J. Morales-Corts M. R. Pérez-Sánchez R. Revilla I. Vivar-Quintana A. M. (2021). Morphometric and nutritional characterization of the main spanish lentil cultivars. *Agriculture* 11:741. 10.3390/agriculture11080741

[B40] Rajiv Kawar P. G. (2016). “Enriched potato for mitigating hidden hunger,” in *Biofortification of Food Crops*, eds Singh U. Praharaj C. S. Singh S. S. Singh N. P. (New Delhi: Springer India), 433–457. 10.1007/978-81-322-2716-8_32

[B41] Reifen R. (2002). Vitamin A as an anti-inflammatory agent. *Proc. Nutr. Soc.* 3 397–400. 10.1079/PNS2002172 12230799

[B42] Rosendaal F. R. (1999). Venous thrombosis: a multicausal disease. *Lancet* 353 1167–1173. 10.1016/s0140-6736(98)10266-0 10209995

[B43] Sab S. Lokesha R. Mannur D. M. Somasekhar Jadhav K. Mallikarjuna B. P. (2020). Genome-wide SNP discovery and mapping QTLs for seed iron and zinc concentrations in chickpea (*Cicer arietinum* L.). *Front. Nutr.* 7:559120. 10.3389/fnut.2020.559120 33154975 PMC7588353

[B44] Sankaran R. P. Grusak M. A. (2014). Whole shoot mineral partitioning and accumulation in pea (*Pisum sativum*). *Front. Plant Sci.* 5:149. 10.3389/fpls.2014.00149 24795736 PMC4006064

[B45] Sharma S. Yadav N. Singh A. Kumar R. (2013). Nutritional and antinutritional profile of newly developed chickpea (*Cicer arietinum* L) varieties. *Int. Food Res. J.* 20 805–810.

[B46] Singh N. P. Pratap A. (2016). “Food legumes for nutritional security and health benefits,” in *Biofortification of Food Crops*, eds Singh U. Praharaj C. S. Singh S. S. Singh N. P. (New Delhi: Springer India), 41–50. 10.1007/978-81-322-2716-8_4

[B47] Solanki D. S. Devi D. D. (2020). Relation between nutrition and immunity. *J. Entomol. Zool. Stud.* 8 2337–2342.

[B48] Sundaram P. Samineni S. Sajja S. B. Roy C. Singh S. P. Joshi P. (2019). Inheritance and relationships of flowering time and seed size in kabuli chickpea. *Euphytica* 215 144. 10.1007/s10681-019-2464-8

[B49] Tefera M. (2021). Study the genetic diversity in protein, zinc and iron in germplasm pools of desi type chickpeas as implicated in quality breeding. *J. Equity Sci. Sustain. Dev.* 4 56–70.

[B50] Thavarajah D. Thavarajah P. (2012). Evaluation of chickpea (*Cicer arietinum* L.) micronutrient composition: biofortification opportunities to combat global micronutrient malnutrition. *Food Res. Int.* 49 99–104. 10.1016/j.foodres.2012.08.007

[B51] Tsai W.-C. Peng Y.-S. Wu H.-Y. Hsu S.-P. Chiu Y.-L. Liu L.-C. (2018). Accuracy of a nutrient database in estimating the dietary phosphorus-to-protein ratio and using a boiling method in low-phosphate hospital diets. *Sci. Rep.* 8:15246. 10.1038/s41598-018-33657-8 30323203 PMC6189135

[B52] van der Maesen L. J. G. (1987). “Origin, history and taxonomy of Chickpea,” in *The Chickpea*, eds Saxena M. C. Singh K. B. (Wallingford: C.A.B. International), 11–34. 10.1016/j.ympev.2021.107235

[B53] Vandemark G. J. Grusak M. A. McGee R. J. (2018). Mineral concentrations of chickpea and lentil cultivars and breeding lines grown in the U.S. *Pacific Northwest. Crop J.* 6 253–262. 10.1016/j.cj.2017.12.003

[B54] Williams P. C. Nakoul H. Singh K. B. (1983). Relationship between cooking time and some physical characteristics in chickpeas (*Cicer arietinum* L.). *J. Sci. Food Agric.* 34 492–496. 10.1002/jsfa.2740340510

[B55] Wood J. Grusak M. (2007). “Nutritional value of chickpea,” in *Chickpea Breeding and Management*, eds Yadav S. S. Redden B. Chen W. Sharma B. (Wallingford: CAB International), 101–142. 10.1079/9781845932138.005

[B56] Zia-Ul-Haq M. Ahmad S. Shad M. A. Iqbal S. Qayum M. Ahmad A. (2011). Compositional studies of lentil (*Lens culinaris* medik.) cultivars commonly grown in Pakistan. *Pakistan J. Bot.* 43 1563–1567.

[B57] Zia-Ul-Haq M. Iqbal S. Ahmad S. Imran M. Niaz A. Bhanger M. I. (2007). Nutritional and compositional study of desi chickpea (*Cicer arietinum* L.) cultivars grown in Punjab, Pakistan. *Food Chem.* 105 1357–1363. 10.1016/j.foodchem.2007.05.004

